# Entrapment of a metal foreign body in the cervical spinal canal during surgical procedure: A case report

**DOI:** 10.1097/MD.0000000000010548

**Published:** 2018-04-27

**Authors:** Xiaoqiang Lv, Xuan Lu, Yue Wang

**Affiliations:** a Department of Orthopedic Surgery, Dongyang People's Hospital, Dongyang; b Spine lab, Department of Orthopedic Surgery, the First Affiliated Hospital, College of Medicine, Zhejiang University, Hangzhou, China.

**Keywords:** anterior cervical corpectomy, cervical myelopathy, cervical spinal canal, retained foreign body

## Abstract

**Rationale:**

Retention of foreign objects in spinal canal usually results from penetrating spinal trauma or failed internal instruments. However, entrapment of a foreign body in cervical spinal canal during surgery is rare, and whether such an object may cause neurological complications remains unknown in literature.

**Patient concerns:**

A 50-year-old man underwent C5 corpectomy and instrumentation surgery due to cervical myelopathy. During the surgery, the cutting edge of a Kerrison rongeur was broken and the metal tip was retained behind C4 vertebra.

**Diagnosis:**

Retention of foreign body in the cervical spinal canal.

**Interventions:**

To remove the metal object, multiple strategies were tried but all failed. As such a metal object was thought to be dangerous to the spinal cord, a remedy C4 corpectomy was performed to remove it. Accidentally, however, the metal fragment further migrated to C2/3 canal. At last, the metal fragment had to be retained in the cervical spinal canal.

**Outcomes:**

At 2-year follow-up, the metal fragment remained in situ and no delayed complications occurred.

**Lessons:**

We reported a rare case of metal object retention in cervical spinal canal due to rongeur fatigue fractures. Under certain circumstances, retention of a small foreign object in spinal canal may not lead to neurological complications. If failed to remove an entrapped foreign body, it may be safe to leave it in the spinal canal for further observation.

## 1 Introduction

Retained foreign objects in the spinal canal, either resulting from penetrating spinal trauma or failed internal instruments, can migrate in the spinal canal and lead to neurological encroachment or some other delayed complications that need further surgical interventions.^[1,2]^ When an instrument is broken and entrapped in the spinal canal during the surgical procedure, immediate removal is recommended.^[2]^ Here, we reported a rare case of rongeur fatigue fractures in an anterior cervical corpectomy surgery, in which the fractured fragment further migrated to proximal cervical spinal canal and was remained there. No further migration and complications occurred in 2-year follow-up.

## 2 Case presentation

**Figure 1:**
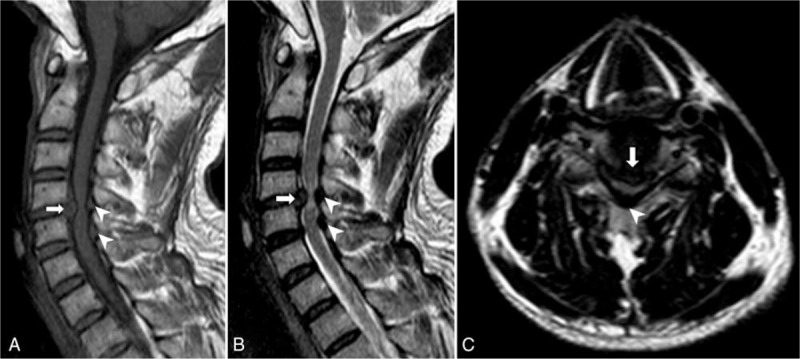
MR imaging showed C4/5 disc herniation (arrow) and thickened ligamentum flavum at C4–5 and C5–6 levels (arrow head), on T1W (A), T2W (B), and axial (C) images. MR = magnetic resonance.

A 50-year-old man presented with neck pain and shoulder pain for 3 months. His pain started 3 months ago, with weakened left arm and finger numbness. One month ago, he had difficulties in walking, including tiredness, numbness, and imbalance. Despite he underwent a variety of conservative therapies, his walking worsened. As such, he was referred to us for surgery. On physical examination, the patient had decreased muscle strength (grade IV) in bilateral deltoids, biceps and triceps, and general dysesthesia over upper limbs. His knee reflexes were hyperactive bilaterally. His Hoffmann sign, Babinski sign, and Romberg sign were positive. Magnetic resonance (MR) imaging revealed C4/5 disc herniation and thickened ligamentum flavum at C4–5 and C5–6 levels, with cervical canal stenosis and local spinal cord compression (Fig. 1).

**Figure 2:**
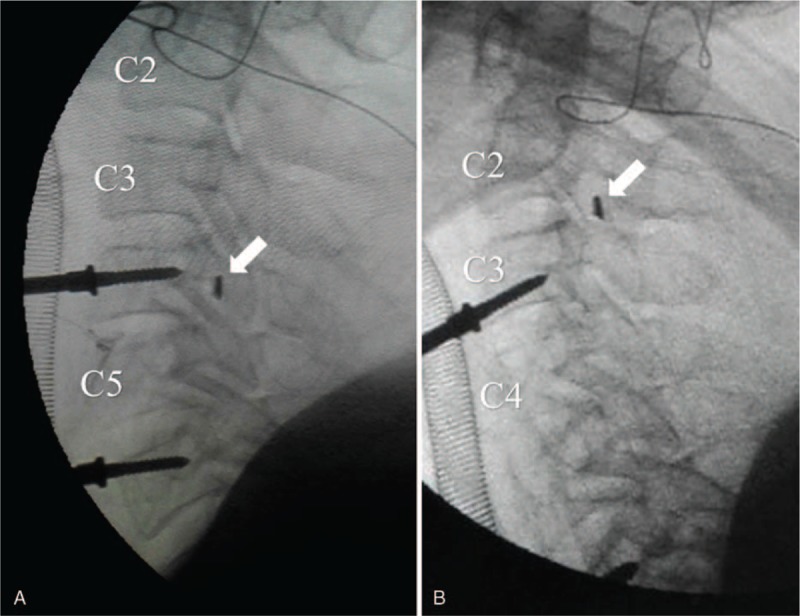
Intraoperative x-ray revealed the broken metal tip (arrow) was located behind C4 vertebral body (A). After C4 vertebral body was removed in the remedy C4 corpectomy, however, the metal fragment was found to migrate to C2/3 canal (B).

The patient was diagnosed as having cervical myelopathy. He underwent C5 corpectomy and decompression surgery in a supine position, with his neck hyperextended. During the surgery, C5 vertebral body was removed and a Kerrison rongeur (blade width 2 mm) was used to remove osteophytes in the posterior edge of C4 vertebral body. Accidently, the cutting edge of the Kerrison rongeur was broken and the broken rongeur tip was missing. As the tip was suspected to remain in the cervical spinal canal, the surgeon immediately searched the anterior epidural space of C4/5 canal using a Penfield hook but failed to find the fractured tip. Intraoperative x-ray showed that the metal tip was behind C4 vertebral body (Fig. 2A). As there was no cerebrospinal fluid leakage, the metal tip was thought to be in the extradural space. Further efforts were made to remove the metal fragment, such as using a longer hook and water washing with a catheter, but all failed.

As such a sharp metal foreign body may migrate with dural pulsation, and penetration into the dural sac may pose the patient to spinal cord injury, the surgical team decided to perform an additional C4 corpectomy to remove the metal fragment. After the majority of C4 vertebral body was carefully removed, however, no metal fragment was found in the epidural space. Further C-arm fluoroscopy revealed that the metal fragment had migrated proximately to C2/3 canal (Fig. 2B). At this stage, further remedy procedures to remove the metal fragment were not practical. After an extensive discussion with the patient's family, the surgeons decided to leave the metal fragment in the cervical spinal canal for next surgery, if necessary. A titanic cage with autograft and an anterior plate were used to fuse C3–6 vertebrae.

**Figure 3:**
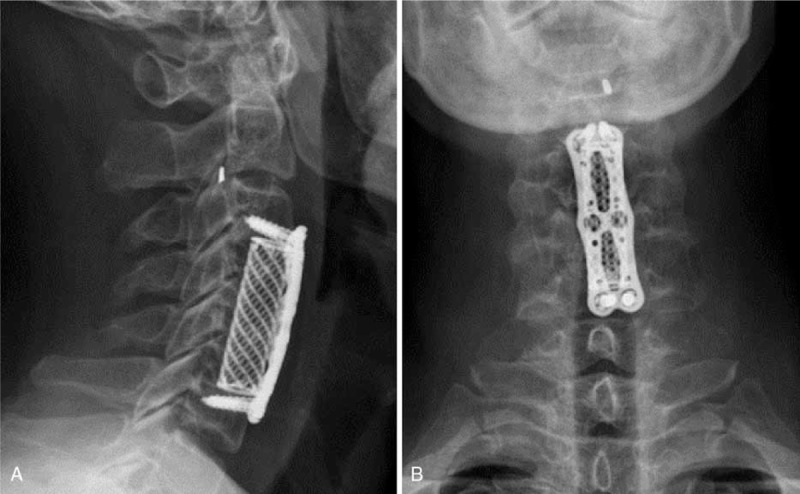
Postoperative x-ray showed the metal fragment was located in the canal immediately behind C2/3 disc (A) and close to left foramen (B).

**Figure 4:**
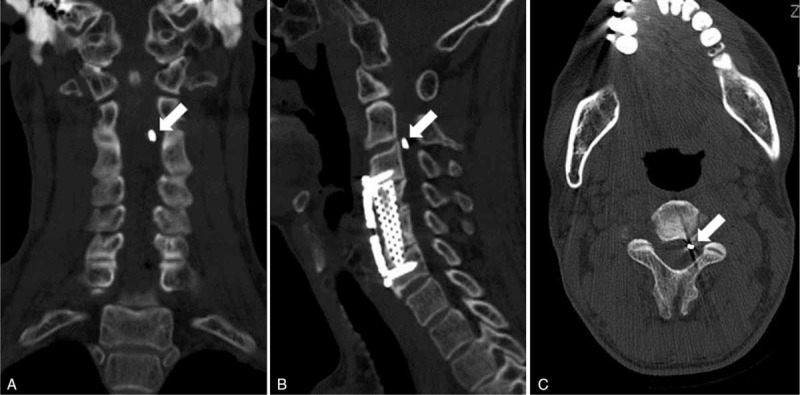
In 2-year follow-up, CT revealed that the metal fragment remained in C2/3 canal and was close to left foramen, without apparent neurological compression. CT = computerized tomography.

After the surgery, the patient's pain disappeared and his neurologic symptoms improved substantially. X-ray showed the fractured metal fragment was in C2/3 canal (Fig. 3A) and close to left foramen (Fig. 3B). The patient returned to normal work and life 3 months after the surgery. In 2-year follow-up, computerized tomography (CT, Fig. 4) showed that the metal fragment remained in situ (C2/3 canal) without apparent neurological compression. The patient had no significant symptoms associated with the cervical spine.

Written informed consent was obtained from the patient for publication of this case report and accompanying images. This case report was approved by ethical committee of Dongyang People's Hospital.

## 3 Discussion

Breakage of instruments during orthopedic procedure reportedly occurs in 0.35% of cases.^[3]^ In spine surgery, it is recommended that such a fractured metal fragment should be removed immediately, as it lies near neurological structures and may migrate into intradural space and cause serious neurological complications.^[3,4]^ This particularly is the case when the fractured instruments are Kirschner wires or cables which tend to migrate.^[3]^ In the spinal canal, the dural sac pulses with heart beats and the dural pressure changes in different postures.^[5]^ As such, there is a “spring phenomenon” which likely pushes foreign body to migrate or penetrate into intradural space, resulting in encroachment upon spinal cord or nerve roots.^[6]^ In our case, the metal piece left in the cervical spinal canal remains in situ without neurological complications in 2-year follow-up. We postulate that granulomas formed around the metal fragment may have restricted it from further migration.

Retention of foreign bodies in the cervical spinal canal is rare. It can be a consequence of failed cervical internal instrumentations^[2,6]^ or penetrating cervical spine injuries.^[7,8]^ In a few reported cases, the retained foreign bodies typically lead to delayed complications within several months or years, including pain, infections, and neurologic dysfunctions.^[9–11]^ In the current case, the foreign body is a fractured rongeur tip. Odds for such a sterile, small but sharp metal object to cause dural sac tear, spinal cord injury, and neurological deficits remain unknown in literature. When the metal fragment was located in the extradural space behind C4 vertebral body, most surgeons would try to retrieve such a “thorn” in the canal. Yet, the metal tip further migrated cranially to C2/3 level during the remedy C4 corpectomy. The reasons for such a secondary migration may include direct push to the rongeur tip in procedure, dural vibration resulting from pulse and surgical manipulations, and gravity effect related to neck hyperextension. If we performed C3/4 discectomy first, and then removed C4 vertebra from cranial endplate down to caudal endplate, the metal tip could have been exposed in surgical field appropriately and the proximal migration be avoided. Yet, we did not expect the metal tip can further migrate proximally at that time.

In the current case, the metal tip is small (2 mm) and no neurological compression occurs in 2-year follow-up. This case offers a piece of evidence that, under certain conditions, small foreign objects in the spinal canal may not always migrate and cause clinical symptoms. However, it remains unclear whether further delayed complications may occur. The patient will be further followed-up.

## 4 Conclusion

We reported a rare case of metal object entrapment in cervical spinal canal during an anterior cervical surgery. Under certain circumstances, retention of a small foreign object in spinal canal may not lead to neurological complications. When failed to remove an entrapped foreign body, it may be safe to leave it in the spinal canal for further observation.

## Author contributions

**Conceptualization:** Xiaoqiang Lv, Xuan Lu, Yue Wang.

**Data curation:** Xiaoqiang Lv, Xuan Lu, Yue Wang.

**Funding acquisition:** Xiaoqiang Lv.

**Investigation:** Xiaoqiang Lv, Xuan Lu, Yue Wang.

**Methodology:** Xiaoqiang Lv, Xuan Lu, Yue Wang.

**Project administration:** Xiaoqiang Lv, Xuan Lu, Yue Wang.

**Supervision:** Xiaoqiang Lv, Xuan Lu, Yue Wang.

**Validation:** Xiaoqiang Lv, Xuan Lu, Yue Wang.

**Writing – original draft:** Xiaoqiang Lv, Xuan Lu, Yue Wang.

**Writing – review and editing:** Xiaoqiang Lv, Xuan Lu, Yue Wang.
